# The Association Between Oxygenation Status at 24 h After Diagnosis of Pulmonary Acute Respiratory Distress Syndrome and the 30-Day Mortality among Pediatric Oncological Patients

**DOI:** 10.3389/fped.2022.805264

**Published:** 2022-05-11

**Authors:** Xueqiong Huang, Lingling Xu, Yuxin Pei, Huimin Huang, Chao Chen, Wen Tang, Xiaoyun Jiang, Yijuan Li

**Affiliations:** Department of Pediatrics, The First Affiliated Hospital, Sun Yat-sen University, Guangzhou, China

**Keywords:** ARDS, oxygenation status, oxygenation index, P/F ratio, risk factors

## Abstract

**Background:**

Pediatric oncology patients with acute respiratory distress syndrome (ARDS) secondary to pneumonia are at high risk of mortality. Our aim was to describe the epidemiology of ARDS in this clinical population and to identify the association between the oxygenation status at 24 h after diagnosis and the 30-day mortality rates, stratified by the severity of ARDS.

**Methods:**

This was a retrospective cohort study of 82 pediatric oncology patients, with a median age of 4 years, admitted to our pediatric intensive care unit with a diagnosis of ARDS between 2013 and 2021. Demographic and clinical factors were compared between the survivor (*n* = 52) and non-survivor (*n* = 30) groups. Univariate and multivariate Cox proportional hazards regression models were used to determine the association between the oxygenation status at 24 h after diagnosis and the 30-day mortality rates.

**Results:**

The mean airway pressure at ARDS diagnosis, PaO_2_/FiO_2_ (P/F) ratio, oxygenation index (OI) value, peak inspiratory pressure, and lactate level at 24 h after ARDS diagnosis, as well as complications (i.e., septicemia and more than two extrapulmonary organ failures) and adjunctive continuous renal replacement therapy, were significant mortality risk factors. After adjusting for other covariates, the oxygenation status P/F ratio (Hazard ratio [HR] = 0.98, 95% confidence interval [CI] = 0.96–1.00, *P* = 0.043) and OI value (HR = 1.12, 95% CI = 1.02–1.23, *P* = 0.016) at 24 h remained independent mortality risk factors. According to the Kaplan–Meier survival curve, a low P/F ratio (≤ 150) and high OI (>10) were associated with a higher risk of 30-day mortality (50.9 and 52.9%, respectively; both *P* < 0.05)

**Conclusion:**

The P/F ratio and OI value measured at 24 h after ARDS diagnosis can provide a better stratification of patients according to ARDS disease severity to predict the 30-day mortality risk.

## Introduction

Acute respiratory distress syndrome (ARDS) is a type of acute-onset hypoxemic respiratory failure that is characterized by the rapid onset of widespread inflammation in the lungs caused by sepsis, pneumonia, trauma, coronavirus disease 2019, and other conditions (1). The prevalence of ARDS in the pediatric intensive care unit (PICU) has been reported to be 3% and is associated with a high mortality rate, ranging between 11 and 61% (2–4). While the management of pediatric ARDS has greatly improved, ARDS among oncological patients in the PICU remains a high-risk factor for mortality. These critically ill pediatric patients frequently require intubation and invasive mechanical ventilation, which are specific risk factors for in-hospital mortality (mortality rates of ~60%) (5, 6).

For a more precise assessment and treatment, one approach to reduce the heterogeneity is to sub-classify patients into those having pulmonary and extrapulmonary ARDS. Pulmonary ARDS accounts for >50% of all ARDS cases (2), while it accounts for 60–80% of all cancer patients (7–9). Pulmonary ARDS has been associated with significantly higher rates of lung epithelial injury, alveolar collapse, fibrin deposition, alveolar wall edema, and lower lung compliance (10, 11). Though some ARDS may be elements of both pulmonary and extra pulmonary injury, there are some different therapeutic management for the two specific subpopulations of patients (12).

In this study, we aimed to describe the epidemiology of pulmonary ARDS in this clinical population and to identify the association between the oxygenation status at 24 h after ARDS diagnosis and the 30-day mortality, stratified by the severity of ARDS. This information will contribute to the development of targeted treatment strategies to improve outcomes of pulmonary ARDS in pediatric oncology.

## Materials and Methods

### Statement of Ethics

Our study protocol was approved by Institutional Review Board of the First Affiliated Hospital of Sun Yat-sen University (number 2021/420, July 1, 2021).

### Study Design and Cohort

**Figure 1:**
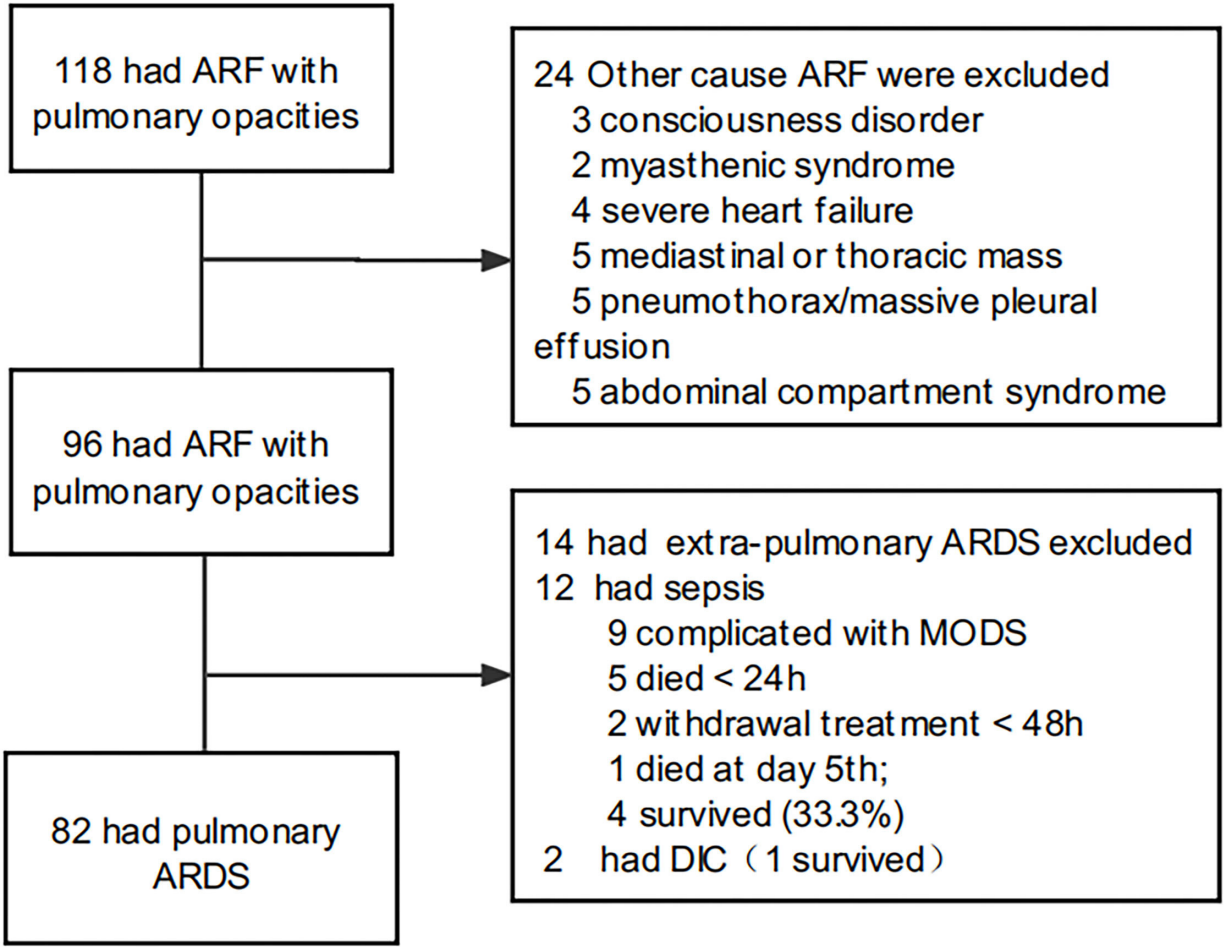
Flow chart concerning ARDS inclusion. ARF, acute respiratory failure; ARDS, acute respiratory distress syndrome.

This was a retrospective cohort study conducted in the eight-bed multidisciplinary PICU of the First Affiliated Hospital of Sun Yat-sen University, between January 2013 and January 2021. In total, 118 oncological children were admitted to the ICU, who had acute respiratory failure with pulmonary opacities, as shown by radiographic or chest computed tomography examination. Among them, the mortality rate of 14 patients with extrapulmonary ARDS was 64.3%. Five patients died within 24 h and two received withdrawal treatment within 48 h after admission. To reduce the heterogeneity, patients with extra pulmonary ARDS were excluded. In total, 82 patients with pulmonary ARDS were included (Figure 1). All patients met the 2015 PALICC meeting criteria and the Berlin criteria for ARDS diagnosis (1, 13). All patients received non-invasive ventilation or invasive mechanical ventilation for >24 h. Patients who died in hospital within 24 h after admission were not included.

### Data Collection

The following data were extracted from patients' electronic medical records for analysis: demographics, clinical data, laboratory tests results, and ventilator parameters [peak inspiratory pressure (PIP), end-expiratory positive pressure (PEEP), mean airway pressure (MAP) and tidal volume]. Laboratory test results included blood gas analysis, routine blood examination, C-reactive protein (CRP) level, procalcitonin level, activated partial thromboplastin time, total bilirubin level, and lactate dehydrogenase level.

The Pediatric Critical Illness Score (PCIS) and the Pediatric Risk of Mortality (PRISM) III score were calculated at the time of admission to the PICU. Severe leukopenia was defined as an absolute white blood count <1,000 cells/mL. The oxygenation index (OI) and the PaO_2_ /FiO_2_ (P/F) ratio were used as markers of the severity of hypoxemic respiratory failure, calculated as follows: OI = (FiO_2_ × MAP × 100)/PaO_2_; and P/F = PaO_2_ /FiO_2_.

### Statistical Analysis

Continuous variables are presented as means ± standard deviations (SDs) or medians and interquartile ranges (IQRs), as appropriate for the data distribution. Categorical data are reported as counts and percentages. Comparisons were made between the survivor and non-survivor groups at 30 days post-admission to the PICU for ARDS.

Baseline characteristics, shown in Table 1, were compared between the two groups using the unpaired two-tailed Student's *t-*test or the Mann–Whitney U test for continuous variables, the unadjusted chi-squared or Fisher's exact test for categorical variables, and the rank-sum test for ranked data. Univariate and multivariate Cox proportional hazard regression models were used to investigate factors associated with the 30-day survival outcome (survivor or non-survivor). The confounder model was adjusted for the P/F (24 h) ratio or the OI (24 h) index, with the following covariates included: age, sex, MAP (0 h), PIP (24 h), lactate (24 h), septicemia, number of extrapulmonary organ failure (≥2), and CRRT. We selected these confounders based on their associations with the outcome of interest (30-day mortality) or a change in the effect estimate >10% on univariate regression analysis. Then, we examined the association between oxygenation status (OI and P/F ratio) at 24 h and 30-day mortality (continuous analysis) and for the non-survivor group specification (dichotomized analysis).

**Table 1:** Comparison of patients' clinical characteristics between the survivor and non-survivor groups.

**Variables**	**Survivors** ***n* = 52**	**Non-survivors** ***n* = 30**	***P*-value**
Age, years	3.90 (1.80–9.40)	4.00 (1.30–7.70)	0.973
Body weight, kg	15.00 (11.10–25.75)	14.50 (7.88–23.62)	0.864
Sex (*n*, male/female)	17/35	11/19	0.715
**Underlying malignancy**			0.076
Leukemia	25 (48.08%)	12 (40.00%)	
Lymphoma	10 (19.23%)	2 (6.67%)	
Solid tumor	16 (30.77%)	12 (40.00%)	
Others	1 (1.92%)	4 (13.33%)	
**Complications**			
Air leak syndrome	3/52 (5.77%)	5/30 (16.67%)	0.109
Abnormal cardiac function	17 (32.69%)	13 (43.33%)	0.335
Acute kidney injury	6 (11.54%)	7 (23.33%)	0.159
Hepatic function impairment	14 (26.92%)	7 (23.33%)	0.72
Cerebral dysfunction	3 (5.77%)	3 (10.00%)	0.479
Septicemia	3 (5.77%)	9 (30.00%)	0.003
Septic shock	6 (11.54%)	9 (30.00%)	0.037
Ventilator-associated pneumonia	6 (11.54%)	5 (16.67%)	0.512
Number of extrapulmonary organ failure (≥2)	8 (15.38%)	12 (40.00%)	0.012
**ARDS severity (0 h, P/F, and PEEP)**			0.179
mild	4 (7.7%)	2 (6.7%)	
moderate	31 (59.6%)	12 (40.0%)	
severe	17 (32.7%)	16 (53.3%)	
**ARDS severity (0 h, OI)**			0.836
≥4, <8	10 (19.2%)	2 (6.7%)	
≥8, <16	23 (44.2%)	15 (50.0%)	
>16	19 (36.6%)	13 (43.3%)	
Ventilation, IMV/NIV	47/5	30/0	0.153
Duration of IMV, days	7.34 ± 4.82	12.17 ± 11.07	0.01
Duration of ICU stay, days	10.0 (6.8–16.0)	12.0 (4.2–18.8)	0.840
Corticosteroid treatment	25/52 (48.1%)	15/30 (50.0%)	0.867
Fluid balance day 0, mL/kg	14.1 (1.9–27.7)	10.7 (−9.0–36.1)	0.606
CRRT	1 (1.9%)	5 (16.7%)	0.023

*Data are reported as means ± SDs, medians (interquartile ranges), or counts (%) as appropriate for the data type. ICU, intensive care unit; IMV, invasive mechanical ventilation; NIV, non-invasive ventilation; CRRT, continuous renal replacement therapy; SD, standard deviation*.

All analyses were performed using Empower(R) (Empower Retirement LLC, Greenwood village, CO, USA) and R (R Foundation for Statistical Computing, Vienna, Austria). Statistical significance was defined as a two-sided *P*-value < 0.05.

## Results

### Clinical Characteristics of the Study Group

The median age was 4 (IQR, 1.5–9.2) years, with a 1:2 male (*n* = 28) to female (*n* = 54) ratio. The most common underlying oncological diagnoses were leukemia (37/82 of patients, 45.1%) and solid tumors (28/82 of patients, 34.1%). The median duration of pre-existing oncological disease was 3 (IQR, 1–8) months. The 30-day non-survival rate was 36.6% (30/82 of patients). Demographic and clinical variables are reported in Table 1. There were no significant differences between the 30-day survivor (*n* = 52) and non-survivor (*n* = 30) groups concerning age, sex, pre-existing comorbid condition, corticosteroid treatment, and fluid balance on day 0 (admission to the PICU). Compared to the survivor group, patients in the non-survivor group had a significantly higher incidence rate of complications, including septicemia, septic shock, and need for adjunctive CRRT therapy (all *P* < 0.05). In this study, six patients received CRRT treatment, four had acute kidney injury, one had cardiac dysfunction, and another one had severe sepsis. In addition, 40% of the patients in the non-survivor group complicated with more than two extrapulmonary organ failures, higher than that in the survivor group (15.38%).

The mortality rate differed based on the criteria used for ARDS diagnosis. However, no significance was found among ARDS severities evaluated at admission between the survivor and non-survivor groups.

### Laboratory Results

Differences in laboratory results between the survivor and non-survivor group are reported in Table 2. Although 21 patients in the whole group had severe leukopenia, there were no significant between-group differences with regard to laboratory findings. The association between the critical illness scores (PCIS and PRISM III), the oxygenation status, and ventilator parameters for the survivor and non-survivor group is reported in Table 3. There were no significant differences of the PCIS and PRISM III scores between the two groups. At the time of ARDS diagnosis, the median OI and P/F ratio values were 15.62 ± 6.50 and 124.71 ± 44.69, respectively, with no difference between the survivor and non-survivor groups. However, at 24 h after diagnosis, the OI values were significantly higher, while the P/F ratio was lower in the non-survivor (27.51 ± 16.6 and 92.51 ± 45.75, respectively) than in the survivor (13.17 ± 7.02 and 160.86 ± 61.85, respectively) group (*P* < 0.001). MAP (0 h), PIP (24 h) and Lac (24 h) were also higher in the non-survivor than in the survivor group (*P* < 0.05).

**Table 2:** Comparison of laboratory results between the survivor and non-survivor groups.

**Variables**	**Survivors** **(*n* = 52)**	**Non-survivors** **(*n* = 30)**	***P*-value**
CRP, mg/L	90.75 (44.80–129.00)	107.90 (11.00–134.00)	0.442
Procalcitonin, ng/mL	0.95 (0.42–5.35)	0.97 (0.34–8.37)	0.761
Creatinine, umol/L	27.50 (19.75–34.25)	36.00 (22.50–77.75)	0.067
Severe leukopenia, n (%)	10/52 (19.2%)	11/30 (36.7%)	0.081
Neutrophils, x10^∧^9/L	2.20 (0.54–4.54)	2.30 (0.16–5.86)	0.402
Lymphocytes, x10^∧^9/L	0.50 (0.23–1.18)	0.28 (0.14–1.56)	0.169
Hemoglobin, g/L	76.00 (65.50–101.50)	79.00 (69.00–90.50)	0.521
Platelet, x10^∧^9/L	78.50 (35.50–173.50)	66.50 (24.00–243.50)	0.766
APTT, s	45.30 (32.85–50.15)	45.20 (35.90–65.80)	0.279
TBIL, umol/L	12.00 (8.30–17.05)	17.35 (12.67–38.70)	0.094
LDH, IU/L	788.00 (480.00–1229.00)	860.00 (695.00–1954.00)	0.051

*All variables were reported as medians (interquartile ranges). CRP, C-reactive protein; APTT, activated partial thromboplastin; TBIL, total bilirubin; LDH, lactate dehydrogenase*.

**Table 3:** The critical illness score, oxygenation status, and ventilator parameters.

**Variables**	**Survivors** ***n* = 52**	**Non-survivors** ***n* = 30**	***P*-value**
**At the time of ARDS diagnosis**			
PCIS (0 h)	77.54 ± 5.98	76.53 ± 5.70	0.458
PRISM III (0 h)	15.83 ± 5.82	18.13 ±5.92	0.090
Lac (0 h), mmol/L	1.00 (0.78–.20)	1.55 (1.02–2.65)	0.269
PIP (0 h), cmH_2_O	23.54 ± 3.83	23.71 ± 2.57	0.835
PEEP (0 h), cmH_2_O	8.93 ± 2.91	8.74 ± 2.52	0.765
MAP (0 h), cmH_2_O	16.80 ± 2.55	18.77 ± 3.42	0.006
Vt (0 h), mL/kg	6.55 ± 1.89	6.72 ± 2.38	0.326
OI (0 h)	15.17 ± 6.26	16.32 ± 6.91	0.454
P/F (0 h)	129.01 ± 44.22	117.25 ± 45.28	0.253
**At 24 h after diagnosis**			
PCIS (24 h)	78.65 ± 5.69	76.53 ± 6.79	0.134
PRISM III (24 h)	16.19 ± 5.69	18.07 ± 5.72	0.155
Lac (24 h), mmol/L	0.90 (0.80–1.30)	1.70 (1.00–4.05)	0.015
PIP (24 h), cmH_2_O	23.93 ± 3.14	26.67 ± 4.88	0.004
PEEP (24 h), cmH_2_O	9.50 ± 2.99	10.87 ± 3.19	0.062
MAP (24 h), cmH_2_O	16.34 ± 2.9	16.46 ± 1.83	0.837
Vt (24 h), mL/kg	6.97 ± 2.19	7.05 ± 2.46	0.125
OI (24 h)	13.17 ± 7.02	27.51 ± 16.63	<0.001
P/F (24 h)	160.86 ± 61.85	92.51 ± 45.75	<0.001

*Data are reported as means ± SDs or medians (interquartile ranges) as appropriate for the data type*.

*PCIS, Pediatric Critical Illness Score; PRISM, Pediatric Risk of Mortality; Lac, lactate; PIP, peak inspiratory pressure; PEEP, end-expiratory positive pressure; MAP, mean airway pressure; Vt tidal volume; OI, oxygenation index; P/F, PaO_2_/FiO_2_ ratio; SD, standard deviation*.

### Association Between Oxygenation (OI and P/F ratio) at 24 h and 30-Day Mortality

**Figure 2:**
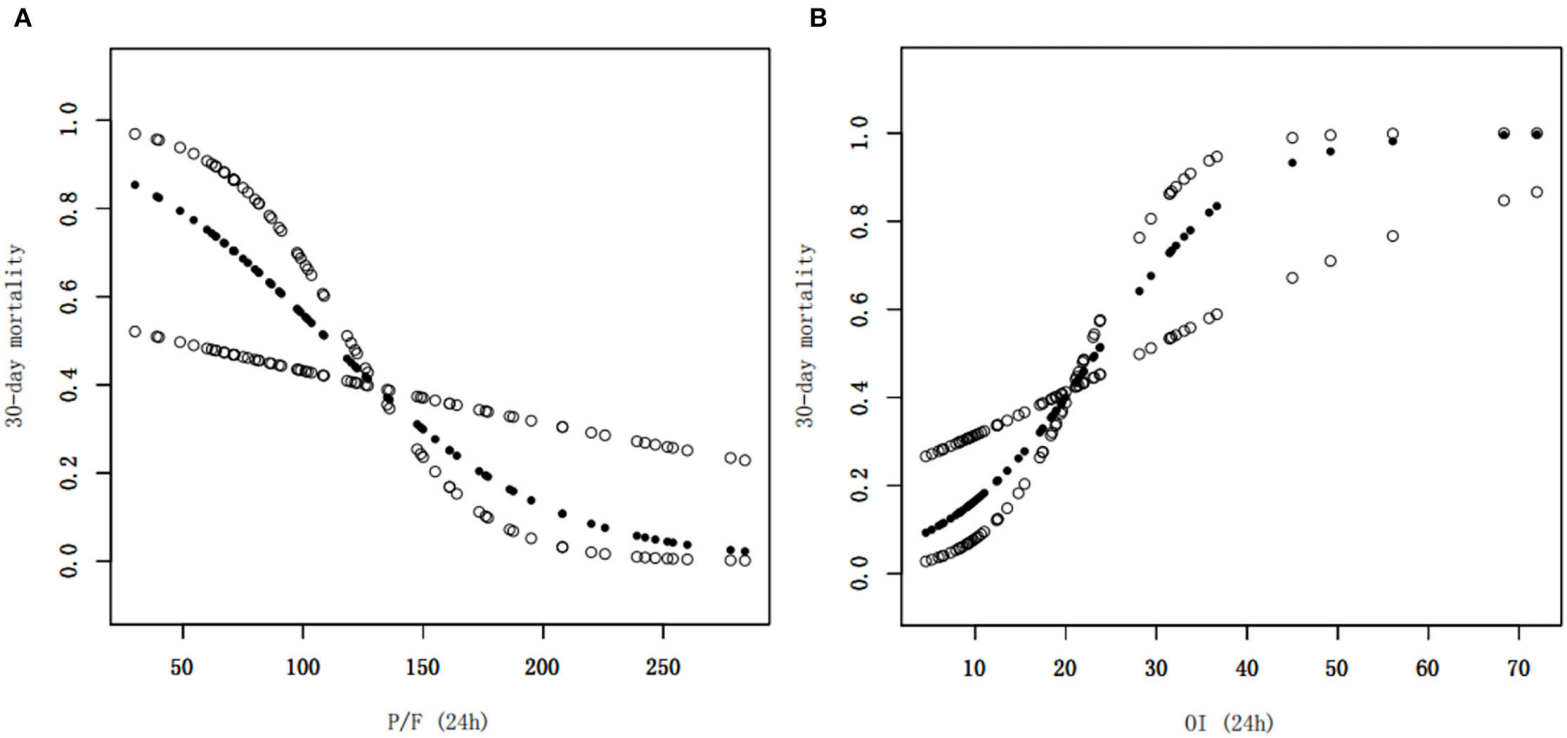
Smoothed regression curves showed that a low P/F ratio and a high OI at 24 h were associated with a higher 30-day mortality risk. The model is adjusted for sex, age, septicemia, MAP (0 h), PIP (24 h), and Lac (24 h), CRRT, and number of extrapulmonary organ failure (≥2). P/F, PaO_2_/FiO_2_; OI, oxygenation index, MAP, mean airway pressure; PIP, peak inspiratory pressure, Lac, lactate, CRRT, continuous renal replacement therapy.

**Figure 3:**
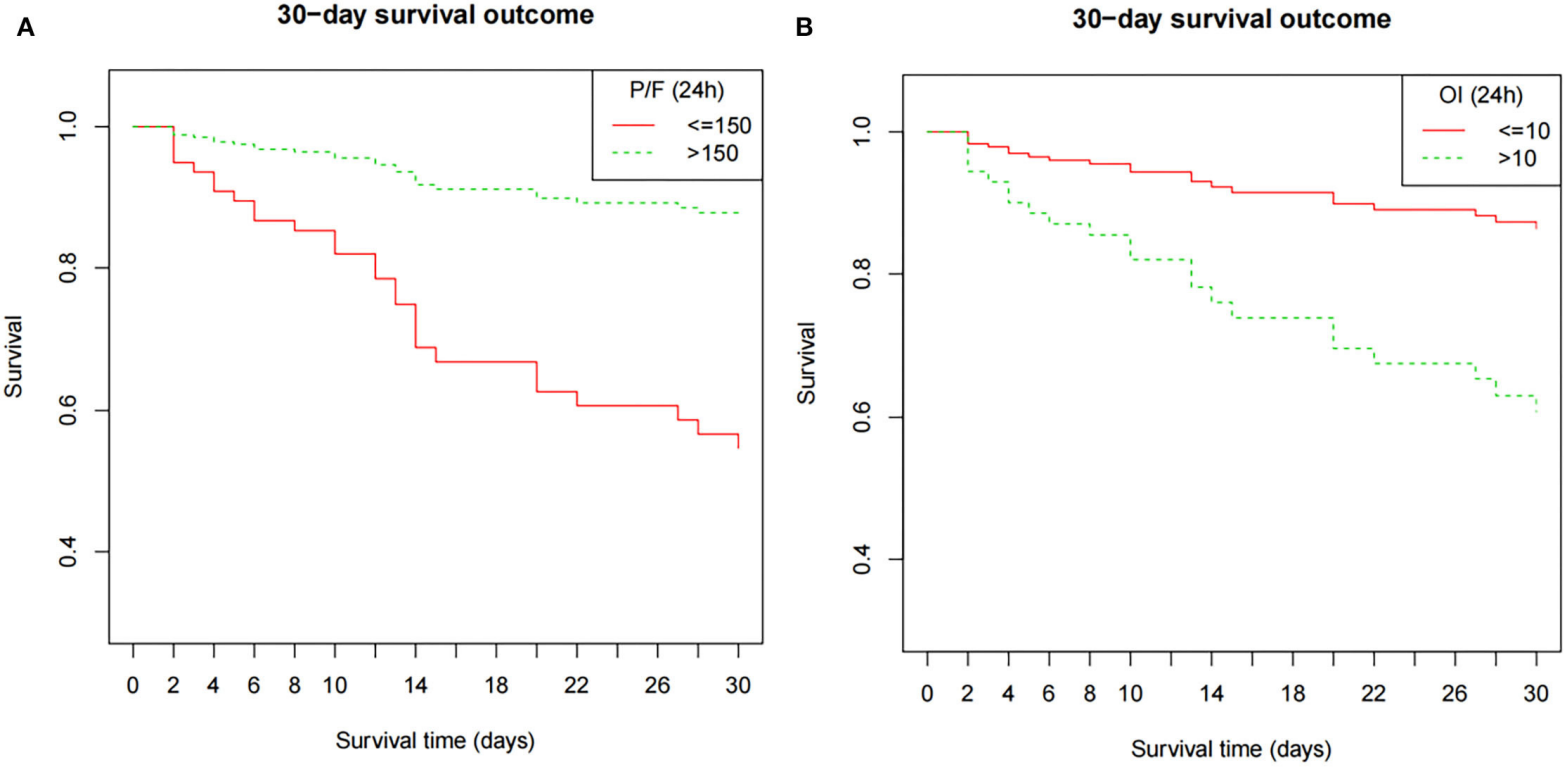
The Kaplan–Meier survival curve as a function of the oxygenation status at 24 h, using dichotomized indices: OI ≥10 and <10; P/F ratio >150 and ≤ 150). The survival results significantly differed between the groups (OI, *P* = 0.047; P/F ratio, *P* = 0.026). The model is adjusted for age, septicemia, CRRT, PIP (24 h), and Lac (24 h). P/F, PaO_2_/FiO_2_; OI, oxygenation index; MAP, mean airway pressure; PIP, peak inspiratory pressure; Lac, lactate; CRRT, continuous renal replacement therapy.

Clinical characteristics and laboratory results were used as independent variables in univariate and multivariate Cox regression models to evaluate the association between the oxygenation status at 24 h and 30-day mortality (Table 4). Both the 24-h P/F ratio and OI were significantly associated with 30-day mortality (Figure 2). After adjusting for other covariates, a low P/F ratio and a high OI at 24 h were associated with a higher 30-day mortality risk (both *P* < 0.05). We further analyzed the risk for 30-day mortality as a function of dichotomized 24 h P/F ratio and OI values for the whole patients, as follows: P/F >150 and ≤ 150; and OI ≥10 and <10). The 30-day mortality rates for severe ARDS reclassified at 24 h were as follows: 50.9% (27/53) for patients with an OI ≥10 and 52.94% (27/51) for patients with a P/F ratio ≤ 150. The 30-day mortality rates in the non-severe group were 10.3% (OI <10) and 9.7% (P/F >150). There was no difference in the duration of mechanical ventilation and ICU stay between the two groups (Table 5). The Kaplan–Meier survival curve analysis confirmed a higher survival rate among patients with a higher oxygenation status at 24 h: OI <10; *P* = 0.047; and P/F >150 (*P* = 0.026; Figure 3).

**Table 4:** Univariate and multivariate Cox regression analyses results of independent variables associated with a 30-day mortality.

**Exposure**	**Univariate**			**Multivariate**		
	**HR**	**95% CI**	***P*-value**	**HR**	**95% CI**	***P*-value**
Septicemia	5.19	1.38, 19.48	0.015	2.33	0.33, 16.50	0.396
Septic shock	2.69	0.86, 8.44	0.090	0.38	0.05, 3.14	0.368
CRRT	13.91	1.44, 134.48	0.023	9.82	0.39, 245.2	0.164
MAP (0 h)	1.25	1.05, 1.49	0.012	0.70	0.40, 1.24	0.221
Lac (24 h)	1.22	1.03, 1.44	0.021	1.02	0.80, 1.29	0.897
PIP (24 h)	1.22	1.05, 1.41	0.008	1.40	0.91, 2.14	0.126
P/F (24 h)	0.98	0.96, 0.99	0.000	0.98	0.96, 1.00	0.043
OI (24 h)	1.14	1.06, 1.22	0.000	1.12	1.02, 1.23	0.016
Number of extrapulmonary organ failure (≥2)	3.18	1.11, 9.08	0.0309	2.59	0.4, 16.94	0.320

*Regression model is adjusted for sex and age*.

*HR, hazard ratio; CI, confidence interval*.

**Table 5:** The association between oxygenation status at 24 h and outcomes.

**At 24h**	**Statistics**	**day mortality** **HR (95% CI)** ***P*-value**	**Duration of IMV (days)** **HR (95% CI)** ***P*-value**	**Duration of ICU stay (days)** **HR (95% CI)** ***P*-value**
OI ≤ 10	29	0	0	0
OI >10	53	−21.87 (−42.09–−1.65) 0.039	−0.21 (−4.13–3.71) 0.916	1.70 (−2.95–6.35) 0.477
P/F ≤ 150	51	0	0	0
P/F >150	31	24.34 (6.39–42.30) 0.010	−0.72 (−4.33–2.89) 0.697	−1.16 (−5.51–3.20) 0.604

*Model is adjusted for sex, age, septicemia, CRRT (24 h), PIP (24 h), and Lac (24 h)*.

*PIP, peak inspiratory pressure, Lac, lactate, CRRT, continuous renal replacement therapy; ICU, intensive care unit; CI, confidence interval; IMV, invasive mechanical ventilation*.

## Discussion

Our study identified a lower oxygenation status at 24 h after ARDS diagnosis as a significant risk factor for 30-day mortality among pediatric oncological patients with pulmonary ARDS. These are important findings considering that acute respiratory failure is a common event in immunosuppressed patients with cancer. Approximately 15% of cancer patients develop acute respiratory failure requiring ICU admission (14). Many studies have demonstrated that oncological diseases are both pre-existing and risk factors for ARDS-related mortality (15, 16). Among adult patients with ARDS, the mortality rate was higher among those with (55.2%) than among those without cancer (24.3%), while it was reported to be 63% in patients with neutropenic cancer at 28 days (9). Chima et al. reported that among pediatric stem cell transplant recipients, only 39% (34/88) of those who required intubation and mechanical ventilation survived in their last PICU admission (17). The total mortality rate in our population was 36.6%. The 30-day mortality rates for severe ARDS reclassified at 24 h were as follows: 50.9% for patients with an OI ≥10 and 52.94% for patients with a P/F ratio ≤ 150, which were slightly lower than those reported in previous studies (7, 8, 18). There are some reasons for it. No patient with graft-vs.-host disease and long-time refractory neutropenia in our study underwent pediatric hematopoietic cell transplantation. Moreover, our cohort comprised only pulmonary cases, associated with less severe comorbidities, such as multiple organ dysfunction syndrome (MODS) and septic shock. However, the incidence of extrapulmonary organ failure (≥2) was higher in non-survivors. Sepsis-related ARDS is commonly associated with higher disease severity and mortality rates compared to non-sepsis-related ARDS. The proportions of sepsis-related ARDS combined with septic shock and MODS were >50 and >80%, respectively (8, 19, 20). In addition, most ARDS reports were focused on adults, especially elderly patients, who had more complications, such as chronic obstructive pulmonary disease, diabetes, chronic cardiac failure, and acute kidney injury (2, 21).

Although all patients in our study had pre-existing comorbid oncological diseases, a uniform treatment strategy was not possible as ARDS is a heterogenous syndrome. The stratification of the severity of ARDS is particularly important for treatment. However, our findings indicate that the oxygenation status (P/F ratio or OI) measured at the time of ARDS diagnosis failed to identify subgroups of patients with a distinct degree of lung injury. It is possible that children with ARDS may not have similar degrees of lung injury or prognosis, although they have a similar oxygenation status at baseline. In addition, some ARDS patients may have improved lung function within the initial 24 h of routine therapy, such as sedation and analgesia, liquid management, antibiotics, and selection of appropriate ventilator parameters. In contrast, oxygenation markers, namely the P/F ratio or OI, measured at 24 h after diagnosis, provides a more precise risk stratification for pediatric oncological patients with ARDS, independent of the patient's septicemia, PIP, and lactate levels. In our study, when ARDS severity was defined by an OI ≥10 or P/F ratio <150, the 30-day mortality rates (50.9 and 52.9%, respectively) were approximately 5-fold higher than those for the non-severe ARDS group (OI <10 or P/F ratio ≤ 150; mortality rates, 10.3 and 9.7%, respectively). More recent studies have reported comparable PICU mortality rates of 55.6 and 58% among children admitted with immunocompromised or oncologic disease requiring mechanical ventilation (18, 22).

Based on our findings, the P/F ratio measured at 24 h after ARDS diagnosis can provide a more precise classification of patients according to ARDS severity. This likely reflects that lung function can improve within the initial 24 h of usual care in some patients with ARDS. Thus, re-stratification of ARDS severity at 24 h after diagnosis would allow identification of patients with non-severe ARDS who would not require more invasive and aggressive therapies. In addition, this re-stratification at 24 h would enable personalized targeted interventions for patients with severe ARDS after 24 h of usual care, including the use of neuromuscular blocking agent (23), corticosteroid therapy (24), prone position ventilation (25), recruitment maneuvers (26), selection of the best ventilatory pattern, and inhaled nitric oxide treatment (27) or extracorporeal membrane oxygenation (28). The proposed re-stratification at 24 h is consistent with autopsy findings reported by Arnaud et al. that the proportion of diffuse alveolar damage depended on the severity of ARDS, being more frequent with moderate and severe ARDS, and that the presence of lung lesions markedly increased at ≥24 h after diagnosis (29).

Although previous studies have demonstrated the inadequacy of initial oxygenation in children and adults with ARDS, relative to oxygenation at 24 h after diagnosis (30–33), our work is the first to focus on a pediatric oncological population with ARDS secondary to severe pneumonia. The association between the oxygenation status at 24 h and mortality was confirmed based on the Berlin and PALICC criteria. However, we did not compare the responsiveness of the OI and P/F ratio for re-stratification of ARDS severity at 24 h after diagnosis. Nevertheless, the distribution of the patients identified relative to the mortality status was similar for the two markers of oxygenation status.

According to lung protection strategies during ventilation, high plateau pressures and tidal volumes should be limited. Panico et al. identified that a higher PIP on day 1 of ARDS was independently associated with mortality in pediatric patients with ARDS (34). Although MAP at ARDS diagnosis and the PIP at 24 h in our study was higher in the non-survivor than in the survivor group, we did not identify a significant correlation between the two factors and the 30-day mortality in the multivariate Cox regression model. This might reflect our use of a relatively balanced PEEP. This result is consistent with those reported by Yehya and Thomas (30) where oxygenation, rather than pressure variables, was the primary metric associated with mortality.

Previous studies have reported higher PRISM III scores and neutropenia as independent predictors of overall mortality among cancer patients with ARDS (18, 20). However, this was not a finding in our study. This may reflect the fact that only patients with ARDS secondary to severe pneumonia were included in our study, rather than other causes, such as sepsis and trauma. Thus, pulmonary ARDS patients had similar PRISM III scores. While our study cohort included 26 patients with severe leukopenia at the time of ARDS diagnosis, leucopenia was not identified as a mortality risk factor in our analysis. It has been suggested that a delay in recovery of neutrophil levels rather than an absolute low level of neutrophils may be associated with poor survival among pediatric oncological patients with acute respiratory failure (35). This variable should be considered in future studies focusing on mortality in this clinical population.

The limitations of our study need to be acknowledged. First, only pediatric oncological patients were included and, therefore, our study cohort was not representative of the entire pediatric population with ARDS. Second, as this was a retrospective study with a limited sample size from a single center, causation cannot be attributed. Therefore, further research is needed.

In summary, pediatric ARDS frequently occurs in oncological children and is associated with a high mortality rate. The oxygenation status at 24 h after diagnosis, classified using the OI and P/F ratio, may provide a better stratification of patients according to ARDS disease severity to predict the 30-day mortality risk. Disease stratification at 24 h could better inform the need for a more aggressive treatment and, in our opinion, should be used for trial enrollment and risk stratification.

## Data Availability Statement

The original contributions presented in the study are included in the article/supplementary material, further inquiries can be directed to the corresponding author/s.

## Ethics Statement

The studies involving human participants were reviewed and approved by Institutional Review Board of the First Affiliated Hospital of Sun Yat-sen University. Written informed consent to participate in this study was provided by the participants' legal guardian/next of kin. Written informed consent was obtained from the minor(s)' legal guardian/next of kin for the publication of any potentially identifiable images or data included in this article.

## Author Contributions

XJ and YL designed the study and reviewed and revised the manuscript. XH and LX carried out the initial analyses and drafted the initial manuscript. YP, HH, CC, and WT coordinated and supervised the data collection. All authors contributed to the article and approved the submitted version.

## Conflict of Interest

The authors declare that the research was conducted in the absence of any commercial or financial relationships that could be construed as a potential conflict of interest.

## Publisher's Note

All claims expressed in this article are solely those of the authors and do not necessarily represent those of their affiliated organizations, or those of the publisher, the editors and the reviewers. Any product that may be evaluated in this article, or claim that may be made by its manufacturer, is not guaranteed or endorsed by the publisher.
